# Respiratory Muscle Strength in Rheumatoid Arthritis

**DOI:** 10.3390/jcm14186455

**Published:** 2025-09-13

**Authors:** Melanie Berger, Maximilian Zimmermann, Leon Thomas, Johannes Strunk, Doreen Kroppen, Daniel Sebastian Majorski, Sarah Bettina Stanzel, Maximilian Wollsching-Strobel, Maxi Schulz, Wolfram Windisch, Falk Schumacher

**Affiliations:** 1 Department of Pneumonology, Cologne Merheim Hospital, Kliniken der Stadt Köln gGmbH, University Witten/Herdecke, 51109 Cologne, Germany; zimmermannma@kliniken-koeln.de (M.Z.); kroppend@kliniken-koeln.de (D.K.); majorskid@kliniken-koeln.de (D.S.M.); stanzels@kliniken-koeln.de (S.B.S.); wollschingstrobelm@kliniken-koeln.de (M.W.-S.); windischw@kliniken-koeln.de (W.W.); 2 Medical Department, University Witten/Herdecke, 58448 Witten, Germany; thomasl@kliniken-koeln.de (L.T.); f.schumacher@khporz.de (F.S.); 3 Department of Rheumatology, Clinic Porz Rhein gGmbH, 51149 Cologne, Germany; j.strunk@khporz.de; 4 Department of Medical Statistics, University Medical Center Göttingen, University Göttingen, 37073 Göttingen, Germany; maxi.schulz@med.uni-goettingen.de

**Keywords:** rheumatoid arthritis, respiratory muscle strength, sarcopenia, handgrip strength, PImax

## Abstract

**Introduction:** Rheumatoid arthritis (RA) is known to affect the musculoskeletal system and, consequently, may lead to sarcopenia, but the role of respiratory muscle involvement in RA patients is unclear. **Methods:** This prospective, exploratory, single-center, matched-pair analysis study was designed to compare respiratory muscle strength and handgrip strength in RA patients and controls. **Results:** RA patients with low disease activity as estimated from the Disease Activity Score 28 (2.3 ± 1.2) and without signs of interstitial lung disease (*n* = 36, 72% female, 28% smoker, mean age 48 + 15 years, mean forced vital capacity 3.9 ± 1.0 L, 98% ± 11% predicted) and control subjects (*n* = 36, 72% female, 11% smoker, mean age 48 + 14 years, mean forced vital capacity 4.1 ± 1.1 L, 98% ± 16% predicted) were well balanced. Maximal inspiratory mouth pressure (PImax, primary endpoint) tended to be lower in RA patients, but this was statistically not significant (−0.9 kPa; 95%CI = −2.11/0.32). However, RA patients more frequently had PImax values below the lower limit of normal (OR 1.74 kPa; 95% CI 0.65/4.77). RA patients had lower handgrip strength (−5.97 kg; 95%CI = −9.43/−2.50). In addition, PImax was correlated to handgrip strength both in RA patients (R = 0.51, *p* = 0.0017) and controls (R = 0.48, *p* = 0.0029) and to the 6-minute walking distance (RA-patients: R = 0.30, *p* = 0.075; controls: R = 0.52, *p* = 0.0012). **Conclusions:** Even though the primary endpoint has not been reached, an impairment of respiratory muscle strength in RA cannot be excluded at least in a subset of patients. Further studies also involving RA patients with more disease activity are needed.

## 1. Introduction

Rheumatoid arthritis (RA) is a systemic inflammatory disease with a progressive chronic course and a prevalence of 0.3–0.8% depending on geographic regions [1]. RA primarily affects the joints, but may also present with extra-articular manifestations, mainly including involvement of the heart and the lungs. However, RA may also cause cachexia [2,3,4] and sarcopenia [5,6]. Importantly, sarcopenia and cachexia are increasingly recognized to be associated with health-related quality-of-life impairments, outcome, and mortality in various chronic diseases [7].

Respiratory involvement has become the center of attention of RA patients in the last decade. This primarily includes interstitial lung disease (ILD), drug-associated lung toxicity, and pulmonary infections [8].

Respiratory muscle strength impairments may primarily occur in different neuromuscular diseases, such as amyothrophic lateral sclerosis, muscular dystrophies, diaphragmatic paralysis, and others [9]. However, sarcopenia and chronic inflammation may theoretically also affect the respiratory system by negatively impacting on respiratory muscle function. As an example, respiratory muscle involvement has been observed in patients with COPD [10] and sarcoidosis [11,12]. Thus, respiratory muscle involvement in RA patients seems to be conceivable.

Of note, the potential impact of RA on respiratory muscle strength has not yet been thoroughly investigated. Based on the lack of knowledge in RA patients, the present study was aimed at assessing respiratory muscle strength in RA patients compared to healthy control subjects.

## 2. Methods

### 2.1. Study Design and Setting

This prospective, exploratory, single-center study was conducted at the Department of Pneumology, Cologne Merheim Hospital, Witten/Herdecke University, Cologne, Germany. Participants were recruited from the pneumonology outpatient clinic. The Ethics Committee for Human Studies, University of Witten/Herdecke, Witten, Germany approved the study protocol for data collection [Proposal no. 195/2021]. Data collection was performed according to the ethical standards set out in the Declaration of Helsinki (last revised: 2013). Written consent was obtained from all participants. The study was prospectively registered in the German Clinical Trials Register (DRKS00027879) and approved on 8th of September 2021 by the Ethics Committee of the University Witten/Herdecke. The STROBE statements for cohort studies were followed when presenting the results of this study.

### 2.2. Data Collection and Patient Characteristics

Data were collected between 10–2021 and 12–2023. Study eligibility was assessed for all patients. RA patients were enrolled according to the American College of Rheumatology/European League Against Rheumatism criteria [13] by consulting rheumatologists. The study design involved a matched-pair analysis to compare RA patients with healthy controls. These were matched for age, sex, and body mass index (BMI).

Patients with chronic alcohol abuse, neuromuscular diseases, chronic obstructive pulmonary disease, sarcoidosis, moderate to severe heart failure, diaphragm palsy, pulmonary hypertension, uncontrolled asthma, diabetes with polyneuropathy, obesity hypoventilation syndrome, and obesity with a BMI ≥ 35 kg/m^2^ were excluded. Patients under 18 years, those with drug abuse, and those with any use of statins or the use of prednisolone >5 mg over a period of 14 days in the last year were also excluded. RA patients with painful or swollen joints of the hands were not recruited. Importantly, none of the recruited RA patients had signs for an ILD as evidenced by unrestricted forced expiratory vital capacity (FVCex; mean 3.9 ± 1.0 L, 98% ± 11% predicted), while velcro crackles were absent in all patients.

### 2.3. Respiratory Muscle Strength Assessment

Volitional tests for respiratory muscle strength including maximal peak inspiratory mouth occlusion pressure (PImax), maximal peak expiratory mouth occlusion pressure (PEmax), nasal Sniff pressure (SnPNa), and mouth occlusion pressure at 100 ms during quiet breathing (P0.1) were measured in accordance with the German recommendations [14]. Participants were instructed to perform maximal inhalations and exhalations from residual volume to total lung capacity and vice versa for PImax and PEmax maneuvers, respectively. SnPNa was measured from functional residual capacity. For maximal pressure assessments, the lower limits of normal have been obtained from the German guidelines for both female and male participants: PImax ≥ 7 kPa (female) and ≥8 kPa (male); PEmax ≥ 7 kPa (female) and ≥10 kPa (male); SnPNa ≥ 6 kPa (female) and ≥7 kPa (male) [14]. PImax, PEmax, and SnPNa are parameters for measuring respiratory muscle strength. SnPNa reflects the strength of the diaphragm, while PEmax and PImax include the whole respiratory muscular apparatus. P0.1 is the airway occlusion pressure at 0.1 s, and it reflects the ventilatory work load and drive of a patient.

### 2.4. General Muscle Strength Assessment

Body muscle strength was estimated from handgrip strength (HGS) of the dominant hand in standing position [15,16], since HGS is also known to correlate with muscle strength in other body compartments [17]. For the purpose of HGS assessment, an electronic hand dynamometer (Jamar^®^ Plus + Dynamometer, Serial Number: 2020120315, made in China, manufactured for: Performance Health Supply, Inc., Warrenville, IL, USA) was used. Maximal values have been obtained, once further increases of HGS could not be achieved anymore.

### 2.5. Pulmonary Function Tests, Diffusion Capacity of the Lungs, and Exercise Testing with Standardized 6-Minute Walk Test

Pulmonary function testing was performed according to the German guidelines [15,16]. A standardized 6-minute walk test (6MWT) was performed in line with recommendations from the American Thoracic Society (ATS) [17], and dyspnea was assessed before and after walking using the modified Borg Scale [18]. Blood gas analysis was measured from the arterialized earlobe immediately before and after the 6MWT. Diffusing capacity of the lung was measured with the single-breath diffusing capacity test in line with recommendations [19].

### 2.6. RA Activity Assessment

RA activity state was assessed through the 28 Disease Activity Score (DAS28) [20,21] with four levels being available: high disease activity >5.1, moderate disease activity >3.2 to <5.1, low disease activity <3.2 to >2.6, and remission <2.6. The results of routine laboratory tests conducted over the preceding six months comprising measurements of C-reactive protein (CRP), creatine kinase (CK), rheumatoid factor (RF), white blood cell count, creatinine levels, anti-citrullinated protein antibody (ACPA) levels, and hemoglobin concentrations were recorded.

### 2.7. Statistical Analysis

The study was planned as a pilot study. Sample-size calculation was performed. Based on a study on a related question, it is conservatively assumed that the average PImax is 12 kPa in rheumatic patients and 14 kPa in non-rheumatic patients, with a standard deviation of 3.0 being assumed for both groups [11]. With an alpha error of 5% and a power of 80%, 36 subjects per group should be included in the study.

Descriptive data are presented as mean ± standard deviation (SD), unless otherwise stated. Statistical tests for group comparisons were selected based on the distribution pattern and variance of the data. For metric characteristics with normal distribution and similar variances, the Student’s t-test was used. In cases of normal distribution with different levels of variance, the Welch’s test was used. For non-normally distributed characteristics with similar variances, the Mann–Whitney U test, and for non-normally distributed data with different variances, the Yuens test was performed. Ordinal characteristics were compared using the chi-squared test (Chi-X^2^). A *p*-value of ≤ 0.05 was considered statistically significant.

Data were analyzed using multiple linear regression to adjust for potential confounders, including age, sex, and BMI. The primary analysis focused on comparing PImax between RA patients and controls. Secondary analyses assessed other pulmonary function parameters diffusion capacity, different values for respiratory muscle function, and exercise tolerance. Data analysis was performed using program R, version 4.3.2.

## 3. Results

Demographic data and smoking status of all participants are listed in Table 1. Smoking status was not matched during recruitment. There was a statistically significant difference (*p* < 0.001), with the control group having more never-smokers and fewer active smokers.

**Table 1:** Demographic data and smoking status.

	RA Patients (*n* = 36)	Controls (*n* = 36)
Female (*n* [%])	26 [72%]	26 [72%]
BMI (kg/m^2^—mean ± SD)	26.0 ± 4.1	25.0 ± 4.0
Body length (m—mean ± SD)	1.7 ± 0.1	1.7 ± 0.09
Body weight (kg—mean ± SD)	73 ± 13	74 ± 13
Age (years—mean ± SD)	48 ± 15	48 ± 14
* Smoking status (*n* [%])		
Never	12 (33)	29 (81)
Active	10 (28)	4 (11)
Quit	14 (39)	3 (8)

BMI = Body Mass Index; RA = Rheumatoid arthritis. * Smoking was not matched: *p* < 0.001. Anti-citrullinated protein antibodies (ACPAs) were <10 U/ml in 16 patients, 12, 16, 46, 107, 169, and 353 U/ml in six others and >500 U/ml in 12 patients with 2 times missing data. Most patients (69%) were in complete remission according to DAS28. Further baseline data that have been assessed specifically for RA patients are given in Table 2.

**Table 2:** Baseline data for Rheumatoid arthritis Patients (*n* = 36).

DAS28—all patients (mean ± SD)	2.3 ± 1.2
DAS28 high disease activity > 5.1 (*n*)	2
DAS28 moderate disease activity > 3.2 to <5.1 (*n*)	4
DAS28 low disease activity < 3.2 to >2.6 (*n*)	5
DAS28 Remission < 2.6 (*n*)	25
Rheumatoid factor /RF (IU/mL—mean ± SD)	48 ± 66
Creatine Kinase/CK (IU/L—mean ± SD)	82 ± 39
C-reactive Protein/CRP < 0.4 mg/dL (n)	33
C-reactive Protein/CRP > 0.4 < 0 1.6 mg/dL (n)	3
Hemoglobin/Hb (g/dL—mean ± SD)	14.6 ± 1.3

DAS28 = Disease Activity Score 28.

Functional parameters are presented in Table 3.

Female patients in the RA group had a PImax of 6.3 ± 2.7 kPa compared to male RA patients (9.6 ± 2.9 kPa). In the control group PImax values were 7.1 ± 2.6 kPa and 10.5 ± 2.4 kPa, respectively. HGS was 24.6 ± 5.2 kg in female RA patients and 44.7 ± 12.4 kg in male RA patients, while HGS were 30.8 ± 5.6 kg and 49,5 ± 9.8 kg in the controls, respectively.

Within the female group of 26 RA patients, the HGS of 9 (35%) patients was below the 10th percentile and 20 (77%) patients below the 50th percentile of the norm. In the RA male patient group, the HGS of 1 (10%) person was below the 10th percentile and 5 (50%) patients below the 50th percentile [22].

Results of multiple regression analyses are presented in Table 4. Here, the primary endpoint was not reached, indicating that global inspiratory muscle strength as estimated from PImax did not significantly differ between the two groups. HGS as a secondary endpoint was significantly affected by RA (*p* < 0.001), and this was also true for the transfer factor for carbon monoxide (TLco) (*p* = 0.03) as presented in detail in Table 4. Other secondary endpoints did not show significant differences (Table 4).

Following binary logistic regression analysis, RA patients more frequently exhibited respiratory muscle function values below the lower limit of normal in comparison to the control group (Table 5).

**Figure 1:**
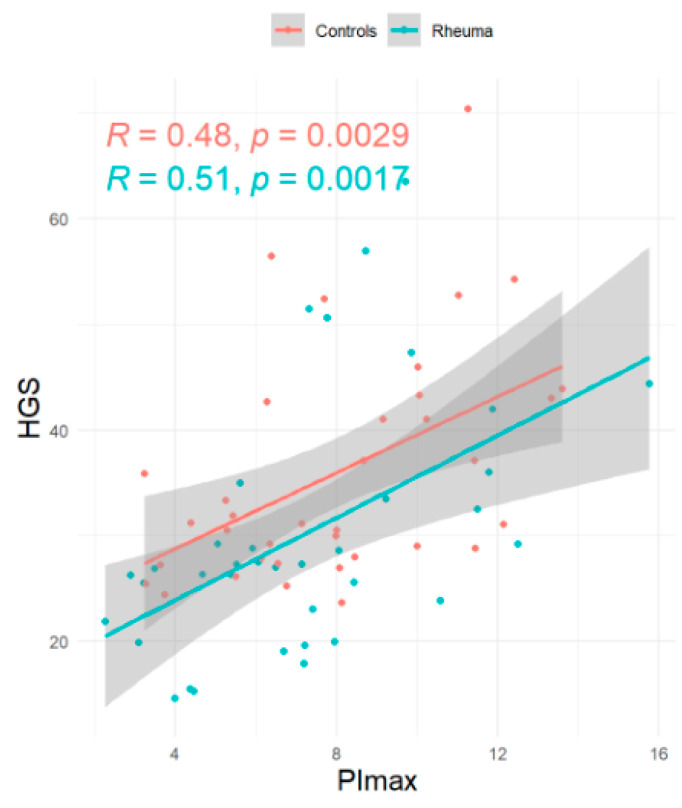
Linear regression: global inspiratory muscle strength (PImax) versus. Handgrip strength (HGS).

**Figure 2:**
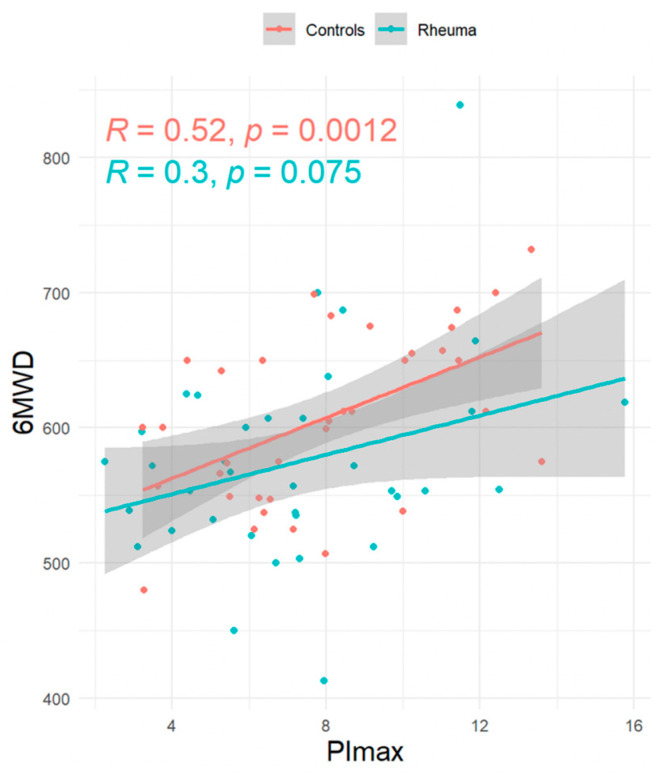
Linear regression: global inspiratory muscle strength (PImax) versus 6-minute walking distance (6MWD).

The association between PImax and HGS is displayed in Figure 1, while the association between PImax and the 6-minute-walking distance (6MWD) is displayed in Figure 2.

## 4. Discussion

This is the first study that has assessed the respiratory muscle strength in RA patients compared to healthy controls using a matched pair analysis. More specifically, the present study has focused on RA patients without signs for ILD, which could have potentially interfered with respiratory pressure assessment following volitional maneuvers as performed in the present study. The main results of the study are as follows: First, the primary endpoint was not met, since PImax did not significantly differ between the two groups, although PImax values were lower in RA compared to the control group. Second, lower limits of normal for respiratory muscle strength were less frequently reached in RA patients compared to the controls, and this was true for PImax, PEmax, and SN PNa. Third, HGS was significantly lower in RA patients compared to controls, and fourth, PImax was correlated to both HGS and 6MWD in RA patients. Reduced HGS in RA patients has been validated before [23], and another study was able to correlate HGS and respiratory muscle strength RA-ILD patients [24].

Based on the current findings, global respiratory muscle function appears to be preserved in RA patients, since the primary endpoint has not been reached. Despite this, the present study also reveals that respiratory muscle dysfunction can, at least, not be excluded in a subset of RA patients, since there was a trend of lower respiratory pressures in RA patients compared to controls. This is also evidenced by the observation that fewer RA patients have reached the lower limit of normal compared to the controls.

Another important result is that female patients in the RA group had a PImax of 6.3 ± 2.7 kPa compared to male RA patients (9.6 ± 2.9 kPa). In the control group, PImax values were 7.1 ± 2.6 kPa and 10.5 ± 2.4 kPa, respectively. HGS was 24.6 ± 5.2 kg in female RA patients and 44.7 ± 12.4 kg in male RA patients, while HGS were 30.8 ± 5.6 kg and 49.5 ± 9.8 kg in the controls, respectively. As expected, female patients in the RA group and females in the control group had a lower PImax and HGS compared to male RA patients and the controls. This result correlates with the literature, where muscle strength is male-dominated [22]. HGS compared to the normal population was also lower in the female RA group, where 35% were below the 10th percentile of the norm and 77% below the 50th. In male RA patients, only 10% were below the 10th percentile and 50% below the 50th. Another study was also able to show that RA patients have lower HGS, with male patients being more affected than female [25]. The results show that gender is an important, yet probably underestimated, factor in rheumatoid arthritis (RA). It is not yet clear whether the gender-related impact is more detrimental for women or men.

The findings of the present study may have clinical implications, although this is limited to assess, until further studies on respiratory muscle function in RA patients are performed. Importantly, RA patients in the present study had low disease activity levels as shown by the DAS28, and most patients had normal CRP values in laboratory assessment, while lung function was reportedly well preserved. Thus, the present results are valid for RA patients with low disease activity. However, respiratory muscle involvement may potentially become even more likely in RA patients suffering from more disease activity or more chronically advanced disease.

It has been clearly acknowledged that sarcopenia is prevalent in the RA in up to 25% [6]. Here, sarcopenia is a muscle disorder that affects the striated muscles and that is progressive over time with the consequence of low muscle strength as a major factor combined with low muscle quantity [7]. Importantly, the respiratory muscles, and, most importantly, the diaphragm, are striated muscles and, thus, sarcopenia may also affect respiratory muscle strength. Interestingly, HGS was reduced in the current RA cohort, which did not suffer from active or even painful arthritis of the hands, indicating that sarcopenia may have been already present at least in some patients. In addition, one study has suggested that respiratory muscles, especially inspiratory muscles, are significantly related to limb muscle strength as assessed by HGS and skeletal muscle mass in geriatric patients [18].

The present study has one major limitation: respiratory muscle strength was only assessed by volitional tests. This, however, means that the results are dependent on the participants performing truly maximal inspiratory and expiratory maneuvers, and this is suggested to be exhausting and unpleasant. In contrast, non-volitional tests for respiratory muscle testing using supramaximal magnetic stimulation of the phrenic nerves are accepted to serve as the gold standard for respiratory muscle testing [14]. Furthermore, the placement of enteral balloon catheters aimed at assessing transdiaphragmatic pressure changes following maximal inspiration are known to further specify diaphragmatic strength [14].

Another limitation was that the study had included well-matched groups (RA patients versus controls), but this was only true for age, sex, and BMI, but not for smoking status. Following, RA patients had been never-smokers much less frequently when compared to the control subjects (33 versus 81%), and this might also explain why diffusion capacity was lower in RA patients (beginning emphysema). Whether this has affected the results remains totally speculative. However, the link between smoking-related chronic obstructive pulmonary disease (COPD) and reduced global inspiratory muscle strength is undisputed [10]. Therefore, it cannot be excluded with certainty that smoking itself has the potential to negatively impact respiratory muscle function, and this should be considered by future studies. Another limitation is the BMI. A more precise reflection of patients’ bodies would have been the body composition, where fat and fat-free mass (muscle, water, and bone) are measured. Also, we only considered oral steroids as a factor for sarcopenia. Other medications were not systematically included.

Finally, PImax values are known to have a substantial interindividual variance that prevents a clear establishment of normal values for PImax [26]. Thus, the sample size of the present study may have been too small to detect true differences in global inspiratory muscle strength as estimated from PImax. Therefore, the failing statistical significance for PImax differences in the present study may not exclude actual differences, which could have been missed due to these methodological issues. More importantly, global inspiratory muscle strength as estimated from the PImax has been shown to be well preserved in patients with ILD due to various conditions, while diaphragmatic muscle strength was shown to be reduced when estimated from non-volitional tests [27]. Following, further studies of RA patients should use non-volitional tests for respiratory muscle strength assessment.

## 5. Conclusions

The present study failed to show a clear reduction in global inspiratory muscle strength in RA patients with low disease activity and also no evidence of ILD. However, the study also shows that impaired respiratory muscle function may be present in a subset of patients as a manifestation of respiratory sarcopenia, and this should be a subject for further studies also of RA patients with more severe disease activity.

## Figures and Tables

**Table 3:** Functional parameters: Rheumatoid arthritis (RA) patients versus control subjects.

	RA Patients (*n* = 36)	Controls (*n* = 36)	Pairwise Comparison Mean Difference (*n* = 36)	Pairwise Comparison 95% Confidence Intervall
PImax (kPa—mean ± SD)	7.2 ± 3.1	8.0 ± 2.9	−0.81 ± 3	[−1.84; 0.21]
PEmax (kPa—mean ± SD)	9.4 ± 4.6	11.4 ± 5.3	−2 ± 6.7	[−4.23; 0.28]
SnPNa (kPa—mean ± SD)	7.9 ± 2.5	8.7 ± 3.0	−0.86 ± 2.9	[−1.84; 0.13]
P0.1 (kPa—mean ± SD)	0.2 ± 0.1	0.2 ± 0.1	0.0 ± 0.17	[−0.06; 0.05]
HGS (kg—mean ± SD)	30.1 ± 11.9	36.2 ± 10.8	−5.8 ± 11.0	[−9.40; 2.28]
6MWD (m—mean ± SD)	574.3 ± 74.9	607 ± 63	−32 ± 89	[−62.62; −1.66]
SpO_2_ before 6MWT (%—mean ± SD)	96 ± 2.7	96 ± 1.3	0.03 ± 2.8	[−0.92; 0.97]
SpO_2_ after 6MWD (%—mean ± SD)	96 ± 1.2	96 ± 1.5	0.18 ± 1.7	[−0.41; 0.78]
FEV_1_ (L—mean ± SD)	3.0 ± 0.7	3.1 ± 0.9	−0.11 ± 0.68	[−0.34; 0.12]
RV (L—mean ± SD)	2.0 ± 0.6	2.0 ± 0.8	−0.15 ± 0.73	[−0.39; 0.1]
Tiffeneau Index (%—mean ± SD)	77 ± 9	75 ± 9	1.7 ± 11.0	[−2.06; 5.56]
FVCex (L—mean ± SD)	3.9 ± 1.0	4.1 ± 1.1	−0.22 ± 0.68	[−0.45; 0.01]
TLC (L—mean ± SD)	5.4 ± 1.3	6.1 ± 1.3	−0.17 ± 1.7	[−0.74; 0.39]
TLco (%predicted)	75.9 ± 13.5	79.5 ± 14	−3.6 ± 20	[−10.35; 3.12]
Kco (% predicted)	83.2 ± 13.3	85.1 ± 14.1	−1.9 ± 20	[−8.57; 4.85]

FVC = forced expiratory vital capacity during expiration; FEV_1_ = forced expiratory volume in 1 s; HGS= handgrip strength; Kco = carbon monoxide transfer coefficient; PImax = maximal inspiratory mouth occlusion pressure; PEmax maximal expiratory mouth occlusion pressure, P0.1 = mouth occlusion pressure at 100 ms; RV = residual volume; SnPNa = nasal sniff pressure; SpO_2_ = peripheral oxygen saturation; TLC = total lung capacity; TLco = transfer factor for carbon monoxide; 6MWD = 6-minute-walk distance; Due to missing data, the following parameters of the pairwise comparison were analyzed in only 35 matched pairs: 6MWD, SpO_2_ before and after 6MWD, and Tiffeneau Index.

**Table 4:** Multiple Regression Analysis with Rheumatoid arthritis as confounder.

	Estimate	2.5%	97.5%	*p* (>[t])
Primary endpoint				
PImax (kPa)	−0.89	−2.11	0.31	0.14
Secondary endpoints				
HGS (kg)	−5.97	−9.42	−2.50	0.001
PEmax (kPa)	−2.00	−4.17	0.15	0.07
P0.1 (kPa)	−0.009	−0.06	0.04	0.99
SnPNa (kPa)	−0.90	−2.05	0.24	0.11
6MWD (m)	−32.31	−65.13	0.70	0.05
FEV_1_ (L)	−0.10	−0.36	0.15	0.42
FVCex (L)	−0.20	−0.50	0.09	0.16
TLC (L)	−0.11	−0.67	0.44	0.68
RV (L)	0.15	−0.22	0.53	0.42
TLco (% predicted)	−0.62	−1.12	−0.05	0.03
Kco (% predicted)	−0.01	−0.11	0.08	0.76

FVC = forced expiratory vital capacity during expiration; FEV_1_ = forced expiratory volume in 1 s; HGS = handgrip strength; Kco = carbon monoxide transfer coefficient; PImax = maximal inspiratory mouth occlusion pressure; PEmax = maximal expiratory mouth occlusion pressure, P0.1 = mouth occlusion pressure at 100 ms; RV = residual volume; SnPNa = nasal sniff pressure; TLC = total lung capacity; TLco = transfer factor for carbon monoxide; 6MWD = 6-minute-walk distance.

**Table 5:** Binary logistic regression analysis for the lower limit of normal [14]: Rheumatoid arthritis patients versus controls.

	OR	2.5% CI	97.5% CI	*p*-Value
Primary endpoint				
PImax (kPA)	1.7	0.65	4.77	0.14
Secondary endpoint				
PEmax (kPA)	2.2	0.77	7.21	0.14
SnPNa (kPA)	1.74	0.65	4.77	0.15

PImax = maximal inspiratory mouth occlusion pressure; PEmax = maximal expiratory mouth occlusion pressure; SnPNa = nasal sniff pressure.

## Data Availability

All data supporting the findings of this study are available within the paper. Further data are available from the authors upon reasonable request. Data are located in controlled access data storage at the Department of Pneumology Clinics of Cologne Germany.

## Abbreviations

ATS—American Thoracic society; ACPA—anti-citrullinated protein antibody; BMI—Body mass index; COPD—Chronic obstructive pulmonary disease; CRP—C-reactive protein; CK—creatine kinase; Chi-X^2^—chi-squared test; DAS28—Disease Activity Score; FVCex—forced expiratory vital capacity; HGS—Handgrip strength; ILD—interstitial lung disease; MWT—Minute walk test; PImax—peak inspiratory mouth occlusion pressure; PEmax—maximal peak expiratory mouth occlusion pressure; P01—mouth occlusion pressure at 100 ms during quite breathing; RF—rheumatoid factor; RA—Rheumatoid arthritis; SnPNa—nasal Sniff pressure; SD—standard deviation; TLco—transfer factor for carbon monoxide.
